# Climate and the Individual: Inter-Annual Variation in the Autumnal Activity of the European Badger (*Meles meles*)

**DOI:** 10.1371/journal.pone.0083156

**Published:** 2014-01-17

**Authors:** Michael J. Noonan, Andrew Markham, Chris Newman, Niki Trigoni, Christina D. Buesching, Stephen A. Ellwood, David W. Macdonald

**Affiliations:** 1 Wildlife Conservation Research Unit, Department of Zoology, University of Oxford, The Recanati-Kaplan Centre, Tubney House, Tubney, Oxfordshire, United Kingdom; 2 Department of Computer Science, University of Oxford, Wolfson Building, Oxfordshire, United Kingdom; Institut Pluridisciplinaire Hubert Curien, France

## Abstract

We establish intra-individual and inter-annual variability in European badger (*Meles meles*) autumnal nightly activity in relation to fine-scale climatic variables, using tri-axial accelerometry. This contributes further to understanding of causality in the established interaction between weather conditions and population dynamics in this species. Modelling found that measures of daylight, rain/humidity, and soil temperature were the most supported predictors of ACTIVITY, in both years studied. In 2010, the drier year, the most supported model included the SOLAR*RH interaction, RAIN, and30cmTEMP (*w* = 0.557), while in 2012, a wetter year, the most supported model included the SOLAR*RH interaction, and the RAIN*10cmTEMP (*w* = 0.999). ACTIVITY also differed significantly between individuals. In the 2012 autumn study period, badgers with the longest *per noctem* activity subsequently exhibited higher Body Condition Indices (BCI) when recaptured. In contrast, under drier 2010 conditions, badgers in good BCI engaged in less *per noctem* activity, while badgers with poor BCI were the most active. When compared on the same calendar dates, to control for night length, duration of mean badger nightly activity was longer (9.5 hrs ±3.3 SE) in 2010 than in 2012 (8.3 hrs ±1.9 SE). In the wetter year, increasing nightly activity was associated with net-positive energetic gains (from BCI), likely due to better foraging conditions. In a drier year, with greater potential for net-negative energy returns, individual nutritional state proved crucial in modifying activity regimes; thus we emphasise how a ‘one size fits all’ approach should not be applied to ecological responses.

## Introduction

In seasonal ecosystems, biological and chemical processes [1], [2], [3], along with phenological responses (e.g., see: [4]), generally respond to annual cyclicity in primary productivity [5], weather patterns, and hydrological, nutrient, and carbon cycles [6], [7], [8]. A species' climatic niche, and range of tolerance around this niche, are thus defining elements in evolution [9], [10], [11]. Considering that ecological responses are typically non-linear [12], [13], even minor changes in environmental conditions can have substantial effects across trophic levels [14]. For consumers, seasonal variation in food supply and associated thermal stresses while foraging create energetic demands [15], [16], [17], driving physiological and/or behavioural adaptations – for example, endocrinological changes [18], fat deposition [19], [20], [21], winter hibernation/torpor [22], [23], life-cycle synchronicity [24], [25], migration [26], and food caching [27], [28]. Matching species' mechanistic responses to fine-scale environmental variability, while imperative, proves challenging (e.g., [29], [30], [31]).

To these ends, recent advancements in accelerometery technology capable of measuring both animal orientation and the dynamics of movement, have considerably enhanced the capacity to achieve behavioural activity sampling on a fine-scale [32], [33], [34], [35]. Accelerometry has elaborated a wide range of behaviours – such as diving [36], [37], feeding [38], [39], and mating behaviour [40] – which often cannot be recorded as systematically by other techniques in wild, cryptic animals [41], [42]. Important in this study, the activity data accelerometers generate can be utilised to provide an accurate proxy for energy expenditure [43].

Here, we apply accelerometry as a tool to investigate the interaction between proximate weather conditions and European badger (*Meles meles*) activity regimes ([44], [13]); that is, this approach enables us to assess how activity is promoted or inhibited by micro-climatic conditions ‘per individual’.

The European badger provides a particularly good model for studying the effects of weather, because of its sensitivity to climatic conditions ([45], [20], [44], [13]) and its trophic specialism, favouring earthworms, *Lumbricus terrestris*, in lowland Britain and Ireland [46], [47], [48], [49].

Badgers undergo autumnal weight gain ([50], [20]) in advance of a flexible extent of winter torpor [51], where they rest inside extensive dens, termed setts [52], [53], [54]. This inactivity period is most evident in higher latitude regions of their range, where winter frosts restrict earthworm availability ([55], [56], [57], [58]). Earthworms become less available under frosty conditions [59], and so in order to maintain or increasing their body-condition in the lead up to food supply restriction (i.e., over the autumn) badgers must attempt to balance trade-offs between the energetic expenditure of their activity (foraging and non-foraging), against energy conserved by inactive periods within their setts. As a consequence early autumn – specifically September – weather conditions present an important transition point in badger activity regimes influenced by variability in the change over from ‘productive’ to ‘less/non-productive’ conditions [20], [44], where more activity has been observed under sub-optimal conditions, to attempt to compensate for lower foraging success [60], [58], [61].

In addition to nutrition, there are also other individual-specific motivators of activity, such as territorial marking [62], [63], scent communication [64], and associated social contact networks [65] potentially pertinent to reproductive success and thus fitness [66]. Consequently, complex individual-specific differences in activity patterns are apparent [67], [68], [69].

Given that interactions between badger population dynamics and weather metrics have been established [20], [44], [13], here we aim to advance understanding causality by examining how variation in autumnal weather conditions in two non-consecutive years influenced activity budgets. We use overall dynamic body acceleration (ODBA) from tri-axial accelerometers [41], [70], to expose real-time variation in badger activity levels through late summer/early autumn. We also postulate that variation in nutritional state will yield differing individual responses to environmental stressors, evidenced as differences in activity levels.

## Methods

The study was conducted on a high-density badger population (36.4 badgers/km^2^, *SE* = 2.55 badgers/km^2^ [71]) in Wytham Woods, Oxfordshire (GPS reference: 51°46′26″N; 1°19′19″W); a 424-ha site of mixed semi-natural woodland (for detailed description of the study site see [20], [72]). As part of a trapping regime undertaken since 1987, badgers were caught in box traps (85×37×38 cm), baited with 150 g of peanuts (but without pre-baiting, making the overall effect of food supplementation inconsequential – see [71]) placed near the entrances of setts and outlying holes (outliers) over two weeks in June (spring), September (summer) and November (autumn), with one week of trapping in January (winter) in some years [71]. Captured animals were transferred to holding cages, between 06:30h and 08:00h the following morning, and transported to a field station using an ATV and trailer. At the field station, individuals were sedated by an intramuscular injection of ketamine hydrochloride (0.2 mL/kg body weight) [73], [74], sexed, measured to the nearest mm (tip of snout to base of sacrum, laid dorsally), and weighed to the nearest 100 g.

From these morphometrics, we used the ratio of body length to weight (L∶W) to calculate Body condition indices (BCI) (log mass vs. log length), as a measure of an individual's nutritional state [75], [76]. To monitor seasonal and individual-specific changes in BCI, we calculated the index for the summers in which animals were instrumented and, provided the animals were (re-)captured, the spring and autumn preceding/following these summers.

We instrumented four badgers in 2010 (3 males and 1 female) and four different individuals in 2012 (3 males and 1 female) with prototype tracking tags [39 mm (l)×22 mm (w)×12 mm (h)] mounted on a leather collar, with a complete assembly weight of 105 g (for detailed description of the collars and data collection system see: [41], [70]). Henceforth individual badgers were identified by their unique collar ID. Tags were equipped with tri-axial accelerometers, sampling pitch, yaw, roll at 8 Hz, as well as radio-frequency identification (RFID), and magneto-inductive sensors (MI) - where a receiver measured the strength of magnetic fields generated by multiple, low frequency loop antennas, allowing the position of the collared animal to be determined in 3-D (see: [41], [70]).

After handling and collaring, badgers were held in a recovery room for 3 h, before being released at the locations where they were captured. Data were compressed and stored onboard the collars, and communicated wirelessly over a 2.4 GHz 802.15.14 link when the animal came above ground, making recapture for data collection unnecessary. In 2010, data were collected from September 28 until batteries ran out on November 24, i.e., for a total of 58 days. All batteries lasted for the same duration except for those in one collar (N30), which ran out after only 45 days. In 2012, data were collected from September 1 until all batteries ran out on October 30, i.e., for a total of 60 days.

We converted these acceleration values for the three axes/channels to overall dynamic body acceleration (ODBA), using the method developed by Wilson et al. [77]. That is, each channel was smoothed individually; using running means over 10 min time frames. The values for these smoothed data, for any particular time frame, were then subtracted from the corresponding unsmoothed data for that time frame. This gives a value for *g* resulting from dynamic acceleration that we converted into absolute positive units using the L1 norm, combining values from all three channels, to produce an ODBA metric. ODBA values were compared to a threshold, determining badger activity from inactivity for each data period (ACTIVITY), (see Appendix S1, Figure S1, Table S1). To satisfy assumptions of normality, hourly measures of activity were subjected to angular transformations for all statistical analyses.

Meteorological records were obtained from the UK Environmental Change Network (ECN)'s weather station in Wytham Woods (T08) and are availably freely. In order to separate the proximate effects of weather on badger ACTIVITY from inter-annual variation, we decomposed climatic data into 7 environmental variables, known to influence badger behaviour and physiology [44], [45], [52], [54]: (1) solar radiation, providing a measure of photoperiod (SOLAR, in W/m^2^) with the additional benefit of including cloud cover and thus more subtle influences on the timing of dusk emergence; (2) relative humidity (RH, in %); (3) mean air temperature (TEMP, in °C); (4) total rainfall (RAIN, in mm); (5) soil temperature at a depth of 10 cm (10cmTEMP, in °C); (6) soil temperature at a depth of 30 cm (30cmTEMP, in °C); (7) volumetric water content of the soil (WATER, m^3^/m^3^). We then modelled the effects of these climatic components on badger ACTIVITY both within each study period and between years.. Given the importance of photoperiod on badger activity [52], we utilised the terms ‘*per diem*’ and ‘*per noctem*’ to distinguish between daylight and night-time hours respectively, and avoid confusion associated with the dual meaning of the term ‘per day’.

### Statistical Analyses

We performed a preliminary analysis of daily measurements using analyses of variance (ANOVA) with analyses of covariance (ANCOVA), paired-sample t-tests, coefficients of variation (CV), and Levene's tests (p = 0.05). As ACTIVITY data were collected over two discrete windows within the two years, analyses relating climate to ACTIVITY were performed for each period separately and then compared.

We then applied an information theoretic approach [78], [79] to examine finer scale-hourly data, using Akaike's information criterion (AIC), in a mixed model approach. SOLAR was included in all multi-variate models to reflect the nocturnality of badgers, and to avoid superfluous testing; no model included more than one measure of temperature (i.e., TEMP, 10cmTEMP, or 30cmTEMP). In addition, because badgers differed individually in their activity regimes, we included individuality as a random effect variable. We acknowledge that although a degree of variation in activity regimes was likely due to inter-sexual variation, sample sizes here did not permit us to investigate this in detail. Given that seven climatic variables were derived for each year, we constructed 26 models without interaction and 33 models that included (an) interaction term(s). We therefore evaluated 59 models with climatic parameters as predictors of badger ACTIVITY. For each model, we derived the AIC, which we used to rank the support for each model (a lower value indicating greater support), as well as the delta AIC (Δ_i_) in relation to the highest ranking model and the Akaike weight (*w*) [78]. As per Anderson [80], Δ_i_ cut-off points were not used, though models with Δ_i_ values above 9–11 were considered to have relatively little support, and models with Δ_i_ values >20 had no empirical support [79]. Statistical analyses were performed using RStudio v. 0.96.331. Although statistical analyses were performed on transformed data, for visual purposes, we used the un-transformed data in all figures.

### Ethics

All trapping and handling procedures were subject to ethical review and were performed under Badger Act (1992) licence (currently 20104655) from Natural England and UK Animals (Scientific Procedures) Act, 1986 licence from the Home Office (currently PPL30/2835).

## Results

### Autumnal Climatic Conditions

Between the autumn study periods in the two years, we found no difference in daily TEMP (ANOVA: F_[1,116]_ = 1.692, p = 0.196), (summarised in table 1), nor between TEMP variability (2010: CV 27.8%, n = 58; 2012: CV = 27.1%, n = 60; Levene's: F_[1,116]_ = 0.662, p = 0.432). Badgers are nocturnal however, and, controlling for night length, 2012 proved significantly colder (daily TEMP 4°C cooler) than 2010 (paired sample t-test: t_32_ = −8.352, p<0.0005) over matched calendar dates.

**Table 1:** Climatic parameters and ACTIVITY.

	2010	2012
TEMP (°C)	11.75±3.24	11.00±2.96
TEMP paired (°C)	13.34±2.11	9.41±2.24
10cmTEMP (°C)	13.07±2.62	12.78±2.38
10cmTEMP paired (°C)	14.65±1.44	11.11±1.38
30cmTEMP (°C)	13.91±1.79	13.90±1.84
30cmTEMP paired (°C)	15.05±0.86	12.47±0.82
RH (%)	83.80±7.43	87.84±8.64
RH paired (%)	83.09±8.48	91.67±6.45
RAIN (mm)	1.82±3.35	2.73±5.75
RAIN paired (mm)	2.21±3.81	2.98±5.68
WATER (m3/m3)	0.306±0.118	0.383±0.053
WATER paired (m3/m3)	0.301±0.117	0.42±0.019
ACTIVITY (hrs)	9.48±3.32	8.27±1.86
ACTIVITY paired (hrs)	11.25±1.02	9.22±1.17

Mean values for climate parameters and ACTIVITY ± SD over the duration of the study periods in 2010 (58 days) and 2012 (60 days). Paired parameters represent means over the same calendar dates (Sep. 28–Oct. 30).

**Figure 1:**
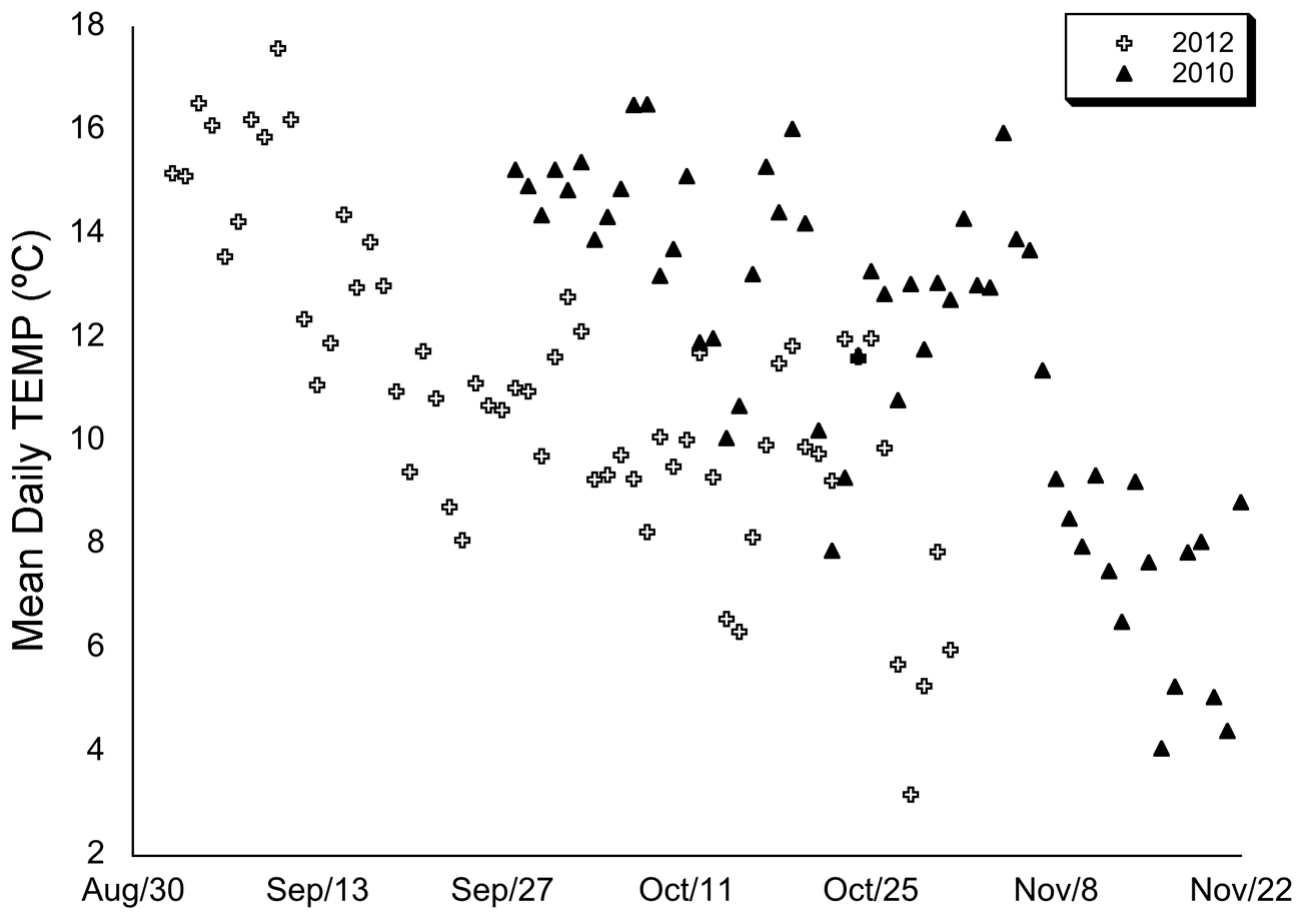
Mean daily TEMP over the study periods. Mean daily air temperature (TEMP) over the course of the study periods in 2010 and 2012. Between the two years, we found no difference in daily TEMP (ANOVA: *F*_[1,116]_ = 1.692, p = 0.196), nor between TEMP variability (2010: CV 27.8%, *n* = 58; 2012: CV = 27.1%, n = 60; Levene's: *F*_[1,116]_ = 0.662, p = 0.432) but 2012 proved significantly colder (daily TEMP 4°C cooler) than 2010 (paired sample t-test: t_32_ = −8.352, p<0.0005) over matched calendar dates.

Decomposing temperature variables further, we found that in 2012 TEMP decreased steadily over the duration of the study period. In contrast, in 2010 there was a cold interlude of 16 days in early November (Nov. 7–Nov. 22), where TEMP decreased rapidly, from 16°C per day to 8.5°C over 5 days, and averaged 7.2°C±1.8 SD over the length of the interlude compared to 13.4°C±1.8 SD over the first 40 days of the study period. TEMP variability over this interlude, through to the end of the study period, was also significantly more variable than the first month and a half of the study (CV = 29.5%, n = 18; CV = 14.9%, n = 40 respectively; Levene's: F[1], [56] = 8.614, p = 0.005) (Fig. 1).

As with TEMP, there was no evidence for a difference in daily 10cmTEMP (ANOVA: F_[1,116]_ = 0.384, p = 0.536), but 2012 was significantly colder than 2010 when compared directly on the same calendar dates (paired sample t-test: t_57_ = 13.546, p<0.0005) with mean daily 10cmTEMP being 3.5°C cooler. Similarly, there was no evidence for a difference in 30cmTEMP, (ANOVA: F_[1,116]_ = 0.003, p = 0.995), but 2012 was significantly colder than 2010 when compared on matched calendar dates (paired sample t-test: t_57_ = 24.707, p<0.0005) with mean per day 30cmTEMP being 2.6°C colder. Within years, 10cmTEMP was significantly more variable than 30cmTEMP (2010: CV = 49.36%, n = 58; CV = 20.21%, n = 58 respectively; Levene's: F_[1,114]_ = 44.499, p<0.0005; 2012: CV = 18.77%, n = 60; CV = 13.41%, n = 60 respectively; Levene's: F_[1,118]_ = 4.781, p = 0.031). Between years, both 10cmTEMP and 30cmTEMP in 2010 were significantly more variable than in 2012 (Levene's: 10cmTEMP; F_[1,116]_ = 51.119, p<0.0005; 30cmTEMP: F_[1,116]_ = 7.011, p = 0.009).

With respect to measures of humidity, although there was no evidence for a difference in RAIN between these years (ANOVA: F_[1,116]_ = 1.089, p = 0.299), the 2012 study period had significantly higher RH (ANOVA: F_[1,116]_ = 7.286, p = 0.008), and WATER (ANOVA: *F*_[1,116]_ = 20.841, p<0.0005) than 2010.

### Badger Activity

#### Inter-annual differences in badger activity

**Figure 2:**
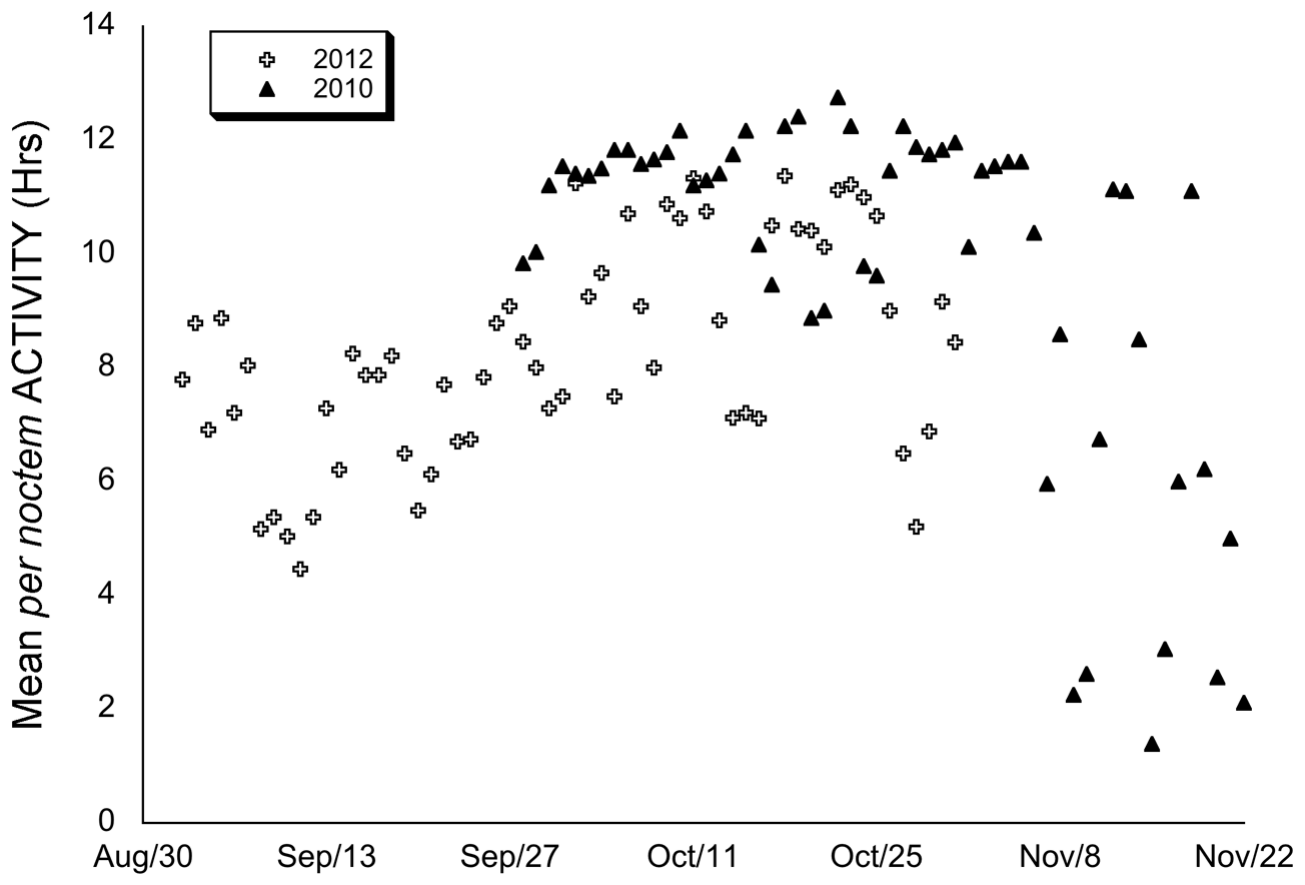
Mean *per noctem* ACTIVITY over the study periods. Mean *per noctem* ACTIVITY over the course of the study periods in 2010 and 2012. The duration of *per noctem* ACTIVITY was significantly more variable in 2010 than it was in 2012 (CV = 35.3%, *n* = 58; CV = 22.7%, *n* = 60 respectively; Levene's: *F*_[1,116]_ = 8.462, p = 0.004), this was also true when compared on the same calendar dates, to control for night length, significantly longer (ANOVA: *F*_[1,1454]_ = 13.510, p<0.0005) in 2010.

From September through October in 2012 *per noctem* ACTIVITY increased with night length and thus correlated negatively with photoperiod (SOLAR: (ANCOVA: F_[1,230]_ = 74.311, p<0.0005). In 2010, however, ACTIVITY was monitored over a later period, from October through November, and *per noctem* ACTIVITY decreased with lengthening nights, resulting in a positive correlation with SOLAR (ANCOVA: F_[1,216]_ = 16.955, p<0.0005). Over the study period in both years, there was no significant difference between individuals (ANCOVA 2010: F_[1,216]_ = 3.301, p = 0.071; 2012: F_[1,230]_ = 3.578, p = 0.060) This suggested that some other variable, such as weather, was counteracting night length. Badgers were significantly more active in 2010 than in 2012 (ANOVA: F_[1,1454]_ = 13.510, p<0.0005) (Table 1), when compared on the same calendar dates between years, to control for night length. As well as being more active, the duration *per noctem* ACTIVITY was significantly more variable in 2010 than it was in 2012 (CV = 35.3%, n = 58; CV = 22.7%, n = 60 respectively; Levene's: F_[1,116]_ = 8.462, p = 0.004) (Fig. 2).

#### Interaction between activity and climatic variables

**Figure 3:**
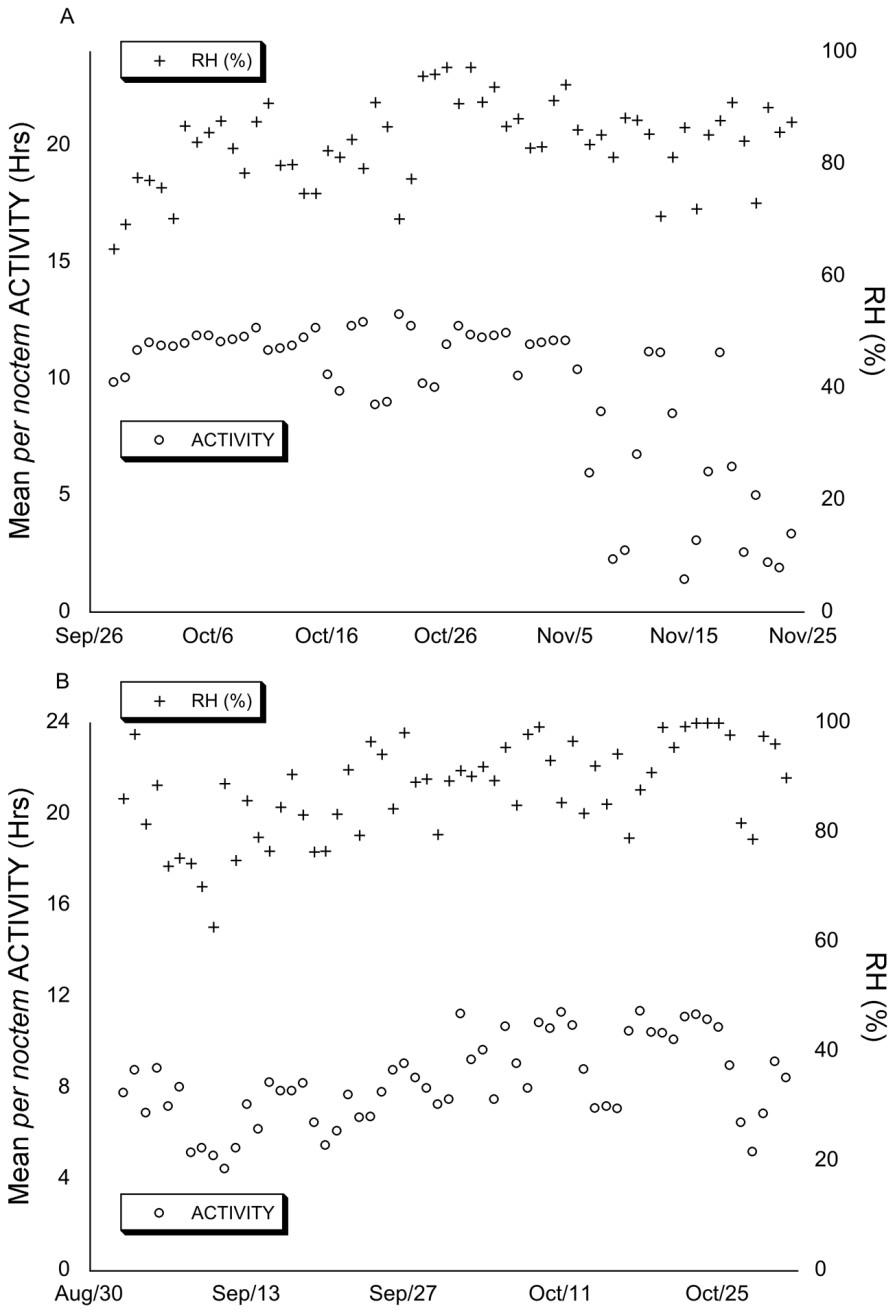
Mean *per noctem* ACTIVITY and RH over the study periods. Mean *per noctem* ACTIVITY and relative humidity (RH) over the course of the study periods in 2010 (A) and 2012 (B). In 2010, there was no correlation between ACTIVITY and RH (F_[1,216]_ = 0.458, p = 0.50). In 2012, ACTIVITY correlated significantly and postively with RH (F_[1,230]_ = 102.593, p<0.0005).

**Figure 4:**
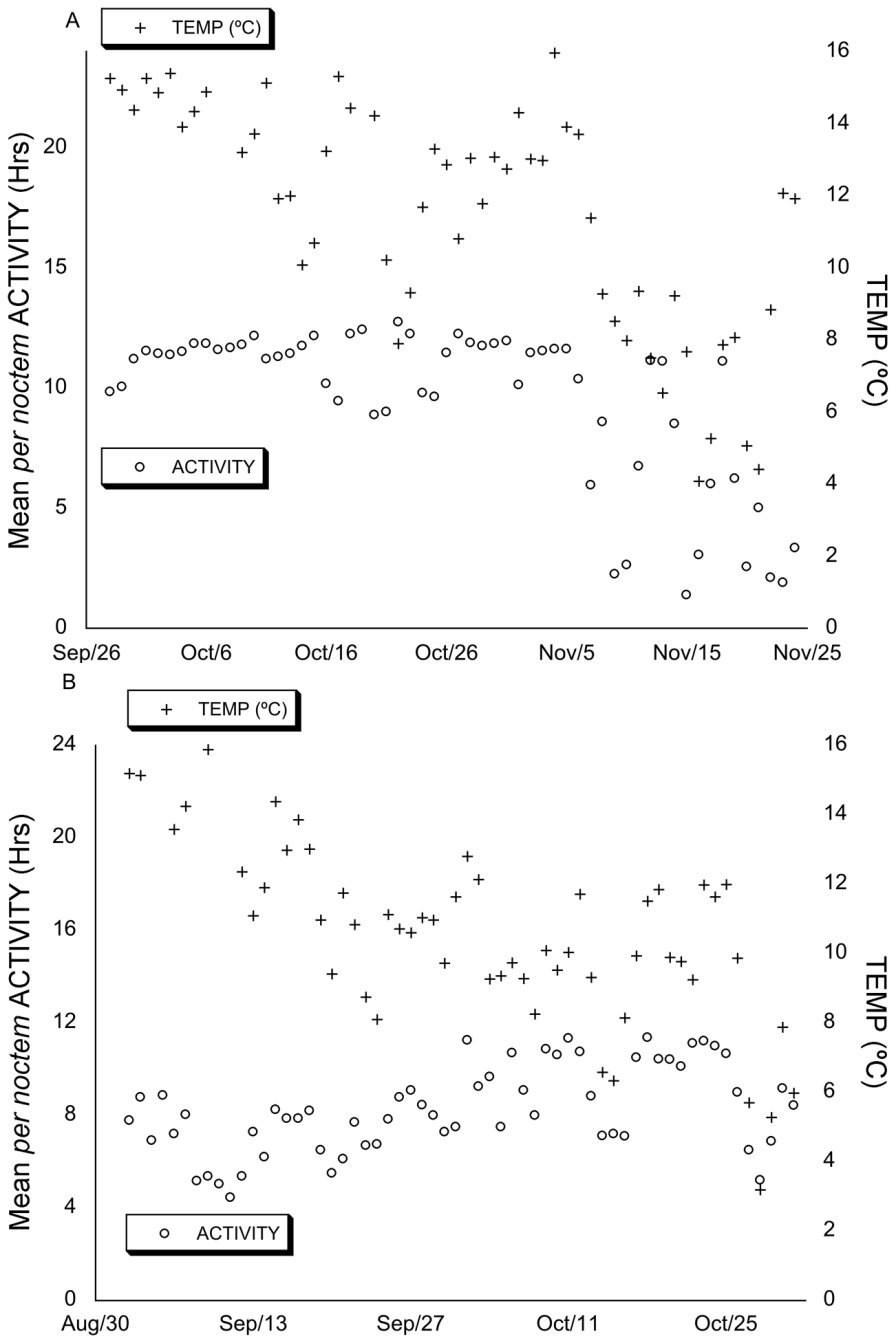
Mean *per noctem* ACTIVITY and TEMP over the study periods. Mean *per noctem* ACTIVITY and daily air temperature (TEMP) over the course of the study periods in 2010 (A) and 2012 (B). In 2010 ACTIVITY correlated significantly and postively with TEMP (F_[1,216]_ = 86.493, p<0.0005), where in 2012, there was no correlation between ACTIVITY and TEMP (F_[1,230]_ = 2.456, p = 0.118).

To explore how the weather conditions influenced *per noctem* ACTIVITY between years specifically, we determined the relationship between the climatic variables and ACTIVITY using ANCOVAs. The study period in 2010 was comparatively drier than 2012, with a pronounced cold interlude, and more variable soil temperatures. In 2010, we observed no correlation between mean badger ACTIVITY and RH (Fig. 3 a) (F_[1,216]_ = 0.458, p = 0.50). There also was no correlation between activity and RAIN (F_[1,216]_ = 0.088, p = 0.77), but a positive correlation between ACTIVITY and TEMP (Fig. 4 a), 10cmTEMP, 30cmTEMP, and WATER (F_[1,216]_ = 86.493, p<0.0005; F_[1,216]_ = 8.942, p<0.005; F_[1,216]_ = 120.510, p<0.0005; F_[1,216]_ = 7.018, p = 0.008 respectively). In all cases, there was no significant difference between individuals (p≥0.063).

As well as being wetter than 2010, soil temperatures were also less variable during the 2012 study period, and we found that ACTIVITY correlated significantly and positively with RH (F_[1,230]_ = 102.593, p<0.0005) (Fig. 3 b), WATER (F_[1,230]_ = 71.524, p<0.0005), and RAIN (F_[1,230]_ = 13.368, p<0.0005). In addition, we found a negative correlation between ACTIVITY and 10cmTEMP, and 30cmTEMP (F_[1,230]_ = 22.205, p<0.0005; F_[1,230]_ = 62.265, p<0.0005 respectively), but no correlation between mean ACTIVITY and TEMP (Fig. 4 b) (F_[1,230]_ = 2.456, p = 0.118). Again, in all cases, there was no significant difference between individuals (p≥0.062).

#### Modelling Activity-climate variable interactions

Informed by our observation that the relationship between these environmental variables and ACTIVITY varied between years, we used information-theoretic models, and finer-scale hourly measures, to ascertain the strongest predictors of *per noctem* ACTIVITY. For 2010, the most supported model included the SOLAR*RH interaction, RAIN, and 30cmTEMP (*w* = 0.557), however a model that included the SOLAR*RH interaction, and the RAIN*30cmTEMP interaction also had some support (*w* = 0.442). Δ_i_ values indicated too much information loss to warrant consideration of other models. For 2012, the most supported model included the SOLAR*RH interaction, and the RAIN*10cmTEMP interaction (*w* = 0.999). Again, Δ_i_ values indicated too much information loss (Table 2) to warrant consideration of other models. Thus, while our uni-variate analyses revealed that the relationship between each environmental variable and ACTIVITY varied inter-annually, this modelling showed that measures of daylight, rain/humidity, and soil temperature were the most supported predictors of ACTIVITY in both years.

**Table 2:** Climatic variables as predictors of *per noctem* ACTIVITY.

2010									
Model	ΔAIC_i_	*w*AIC_i_	β_0_	*var*_(INDIVIDUAL)_	β_SOLAR*RH_	β_SOLAR_	β_RH_	β_Temperature_	β_RAIN_
SOLAR*RH+RAIN+30cmTEMP^a^	0	0.557	−46.78 (3.94)	0.14%	−0.002 (<0.001)	0.070 (0.01)	0.24 (0.03)	4.39 (0.18)	−6.12 (0.88)
SOLAR*RH+RAIN*30cmTEMP	0.46	0.442	−46.25 (3.97)	0.63%	−0.002 (<0.001)	0.071 (0.01)	0.24 (0.03)	4.35 (0.18)	−13.44 (6.84)
SOLAR*RH+30cmTEMP	47.18	<0.001	−43.91 (3.94)	0.13%	−0.002 (<0.001)	0.065 (0.01)	0.19 (0.03)	4.41 (0.18)	-
SOLAR*RH+RAIN*10cmTEMP	124.32	<0.001	−22.82 (3.56)	0.63%	−0.002 (<0.001)	0.069 (0.01)	0.28 (0.03)	2.60 (0.12)	−7.19 (0.90)
SOLAR*RH+RAIN+10cmTEMP	126.75	<0.001	−23.36 (3.55)	0.14%	−0.002 (<0.001)	0.065 (0.01)	0.26 (0.06)	2.58 (0.19)	−7.11 (1.55)
SOLAR*RH+10cmTEMP	190.68	<0.001	−19.14 (3.53)	0.14%	−0.002 (<0.001)	0.062 (0.01)	0.23 (0.03)	2.55 (0.19)	-
SOLAR*30cmTEMP	227.83	<0.001	−42.87 (3.01)	0.71%	-	0.058 (0.02)	-	5.46 (0.21)	-
SOLAR+RAIN+30cmTEMP	254.46	<0.001	−28.59 (2.59)	0.13%	−0.002 (<0.001)	−0.099 (0.002)	-	4.49 (0.18)	−5.39 (0.89)
SOLAR+RH*30cmTEMP	272.53	<0.001	62.69 (18.00)	0.24%	-	−0.102 (0.002)	−1.09 (0.21)	−1.26 (1.23)	-
Intercept	2710	<0.001	24.28 (0.38)	-	-	-	-	-	-

2012									
Model	ΔAIC_i_	*w*AIC_i_	β_0_	*var*_(INDIVIDUAL)_	β_SOLAR*RH_	β_SOLAR_	β_RH_	β_Temperature_	β_RAIN_
SOLAR*RH+RAIN*10cmTEMP^a^	0	0.999	−9.74 (3.84)	0.23%	−0.002 (<0.001)	0.08 (0.01)	0.29 (0.03)	0.98 (0.13)	48.89 (5.76)
SOLAR*RH+RAIN*30cmTEMP	17.1	<0.001	−5.01 (4.22)	0.23%	−0.002 (<0.001)	0.08 (0.01)	0.26 (0.03)	0.77 (0.17)	64.46 (6.91)
SOLAR*RH+TEMP*WATER	58.24	<0.001	19.90 (8.69)	0.23%	−0.002 (<0.001)	0.07 (0.01)	0.25 (0.03)	−1.75 (0.70)	-
SOLAR*RH+RAIN+10cmTEMP	67.89	<0.001	−7.45 (3.86)	0.23%	−0.002 (<0.001)	0.08 (0.01)	0.28 (0.03)	0.83 (0.13)	0.96 (0.58)
SOLAR*RH+RAIN*WATER	68.21	<0.001	8.19 (3.48)	0.22%	−0.002 (<0.001)	0.08 (0.01)	0.23 (0.03)	-	−19.51 (3.81)
SOLAR*RH+10cmTEMP	69.38	<0.001	−8.00 (3.84)	0.23%	−0.002 (<0.001)	0.08 (0.01)	0.29 (0.03)	0.82 (0.13)	-
SOLAR*RH+TEMP+WATER	70.67	<0.001	−6.33 (6.93)	0.23%	−0.002 (<0.001)	0.07 (0.01)	0.27 (0.03)	0.59 (0.10)	-
SOLAR*RH+TEMP*RAIN	78.74	<0.001	−1.69 (3.49)	0.23%	−0.002 (<0.001)	0.07 (0.01)	0.28 (0.03)	0.54 (0.10)	-
SOLAR*RH+TEMP	78.89	<0.001	−2.25 (3.46)	0.23%	−0.002 (<0.001)	0.07 (0.01)	0.28 (0.03)	0.55 (0.09)	-
Intercept	1918	<0.001	20.38 (0.36)	-	-	-	-	-	-

^a^ The selected model.

Model selection using Akaike's information criterion (AIC) to assess variation in hourly ACTIVITY in 2010, and 2012. The parameter β_0_ provides an estimate of the model intercept of the angular transformed ACTIVITY in a linear function, with standard error in parentheses. In addition, *var*_(INDIVIDUAL)_, an estimate of the variation between individuals for each climatic prediction model, and parameter estimates for each model are also presented; as all models included only one measure of temperature, β_Temperature_ refers to the parameter used (i.e., TEMP, 10cmTEMP, 30cmTEMP). Akaike differences (ΔAIC), and weight (*w*AIC_i_) relative to the best model are also provided. For clarity, only the top 10 models in each year are listed, including the intercept.

### Individual Differences in Badgers Activity Regimes

Within this climatic framework, in both years ACTIVITY also differed significantly between individuals (ANOVA: F_[3,5280]_ = 4.866, p = 0.002; F_[3,5496]_ = 3.382, p = 0.017 respectively). We therefore compared BCI, as a proxy of nutritional state, against individual activity regimes, as well as the population mean (PM) values for BCI ± the standard deviation (SD).

**Figure 5:**
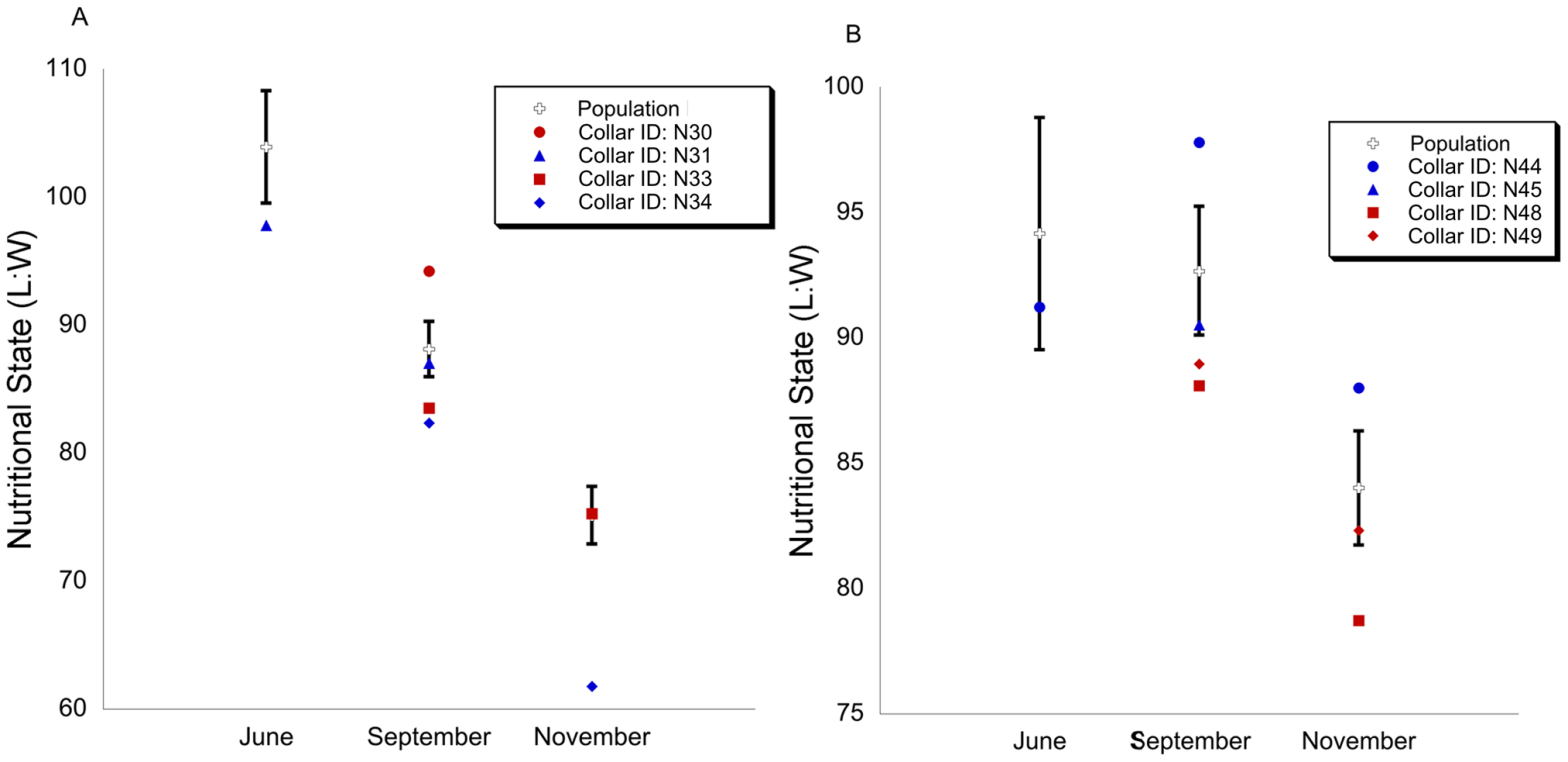
Variation in individual nutritional states from the population. Individual measures of nutritional state (L∶W) in comparison to the population mean (PM) ± the standard deviation (SD) in June, September, and November in 2010 (A) and 2012 (B). Red points indicate badgers with comparatively long ACTIVITY, where blue points indicate badgers with comparatively short ACTIVITY.

In 2010, badgers with higher BCI, exhibited less *per noctem* activity than badgers with lower BCI. When first captured in September, only one badger (N30) had a significantly poorer BCI than the PM. This individual also exhibited the longest mean ACTIVITY duration *per noctem* compared to all other badgers tracked that year, being active on average 30 min longer than conspecifics; this greater activity was statistically significant when compared to badgers N31 and N34 (Tukey *post-hoc*: p = 0.003; p = 0.020 respectively). N31 and N34 had BCIs significantly higher than the PM. N33 was, on average, also active for 30 min longer *per noctem* than N34, however this was not statistically significant (ANOVA: F_[1,2789]_ = 2.801, p = 0.094). When recaptured in November, N33 had a lower BCI than N34, and was within the PM, while N34's BCI had increased, and was still well above the PM (Fig. 5a).

In contrast in 2012, badgers with the longest *per noctem* activity exhibited higher BCIs when recaptured in November. BCI for N48 and N49 both exceeded the PM when collared in September. When recaptured in November, only N48 remained above the PM, and had been more active (on average 20 minutes longer *per noctem*) than N49, for which BCI had fallen to within the PM. Badger N44 had a lower BCI than both N48 and N49, and was below the PM in both September and November. On a *per noctem* basis, N44 was, on average, active for 40 min less than N48 and 20 min less than N49. Badger N45 was the least active over the course of the study period, and was within the PM when collared in September, however it was not recaptured in November, and so no conclusions can be drawn for that individual concerning autumnal changes in BCI (Fig. 5b).

## Discussion

Badgers are responsive to climatic conditions [45], [20], [44], [13] and, in this study population, autumnal conditions have been found to present a critical period for all aspects of badger population dynamics [44], testing the ability of badgers to balance energy budgets. Applying fine-scaled measures of activity allowed us to determine with high resolution that individual badger activity regimes varied in response to proximate weather, and with respect to body-condition. These clear inter-annual differences in the way individuals responded to varying climatic conditions evidence that biological responses, phenology and cyclicities in annual physiological and behavioural regimes should not be assumed to follow the same course each year.

Although there are various factors that can influence badger activity (for example Göransson [81] found that, in autumn, badgers spent over 2–5 h daily digging and collecting material for bedding), our modelling indicates that interactions between solar radiation and humidity, rainfall, and soil temperatures were significant drivers of badger *per noctem* autumnal activity.

In relation to Nouvellet et al's [13] observation that survival rates decline outside of the typical annual range of daily predicted temperature norms in this population, we found that badgers were significantly more active in the drier year (2010) than a wetter one (2012).

Furthermore, we identified the generality for those badgers attaining the highest BMI to also be the most active individuals in 2012; the wetter of the two years. This implies that whatever specific activities contributed to their nightly regime, foraging must have being among these, and returned net-positive energetic gains. In 2010, with drier conditions, we observed a more subtle response: ‘thinner’ badgers (BCI<PM) were more active than ‘fatter’ badgers (BCI>PM).

Given that badgers in this region [46], [47], [48], [49], [82], as well as in others (e.g., [83], [58]), favour earthworm prey (see above), and given that earthworms surface and become most available to predators under mild, moist microclimatic conditions [57], [84], [58], this syllogism leads us to strong inference that activity relates in major part to foraging success. Badgers with higher BCI prove freer to obviate the risk of net-negative energy returns under less optimal conditions by reducing their nightly activity levels.

Our tri-axial accelerometry results resonate with other studies that report increased badger foraging-related activity under sub-optimal climatic conditions (e.g., in Spain [60], Poland [58], and Switzerland [61]). Under conditions that limit earthworm availability (i.e., below 2°C: [55], [56], [57], [58]), Kowalczyk et al. [58] report that badgers attempt to compensate by devoting over 2.5 hrs more to foraging. In addition, maintaining body-temperature is more energetically expensive when active in cold, wet conditions [85], [86] – see also [44], [13]. This defines a suite of optimal weather conditions that can influence badger foraging success [87], ability to improve/maintain body condition [20] and thus ultimately survival probability [44], [13].

Based on the inter-individual differences in activity regimes we observed, we emphasise that a ‘one size fits all’ approach cannot be applied to ecological responses. State-dependent risk-taking is well documented across a diversity of taxa [88], where changes in behaviour to maintain positive energy budgets have been linked to climate variability (e.g., caribou (*Rangifer tarandus*) [89]; badgers [44]; Eurasian beavers (*Castor fiber*) [4]). According to Clark [90], individuals with greater reproductive value - a function of body condition in badgers [91], [20], should increase risk aversion in comparison to individuals with less reproductive value, in order not to jeopardise survival and future reproduction. Conversely, some individuals will take greater risks to achieve future fitness returns than individuals with greater reproductive value [92]. We infer that predictable conditions seem to be important for optimal survival dynamics, allowing individual badgers to prospect risk most effectively [93] and optimise their annual routines [94].

In conclusion, these accelerometry data further advance understanding of badger-climate interactions, with detail allowing us to begin to dissect the causal mechanisms involved. This level of resolution is essential – beyond identifying indicative trend relationships – in order to identify the ecological consequences of weather patterns (or indeed other influential factors in other systems) and understand individual-specific adaptive responses, in turn better informing ability to devise conservation policy [95].

## Supporting Information

Figure S1 **Perceived activity as a function of the threshold value.** Percent active as a function of the threshold value (T) for badger N44 over the course of the study period. The threshold value chosen for this individual was 10.
(TIF)

Table S1 **Perceived hourly activity.** Hourly activity (in minutes) for four collared badgers over the study period in (A) 2010 and (B) 2012. For queries on finer-scale data please contact the corresponding author.
(XLSX)

Appendix S1 (DOCX)
