# Supplementary figures and images for: Climate and the Individual: Inter-Annual Variation in the Autumnal Activity of the European Badger (Meles meles)
Source: PLoS One. 2014 Jan 17;9(1):e83156. doi: 10.1371/journal.pone.0083156 (PMC3894934; doi:10.1371/journal.pone.0083156)

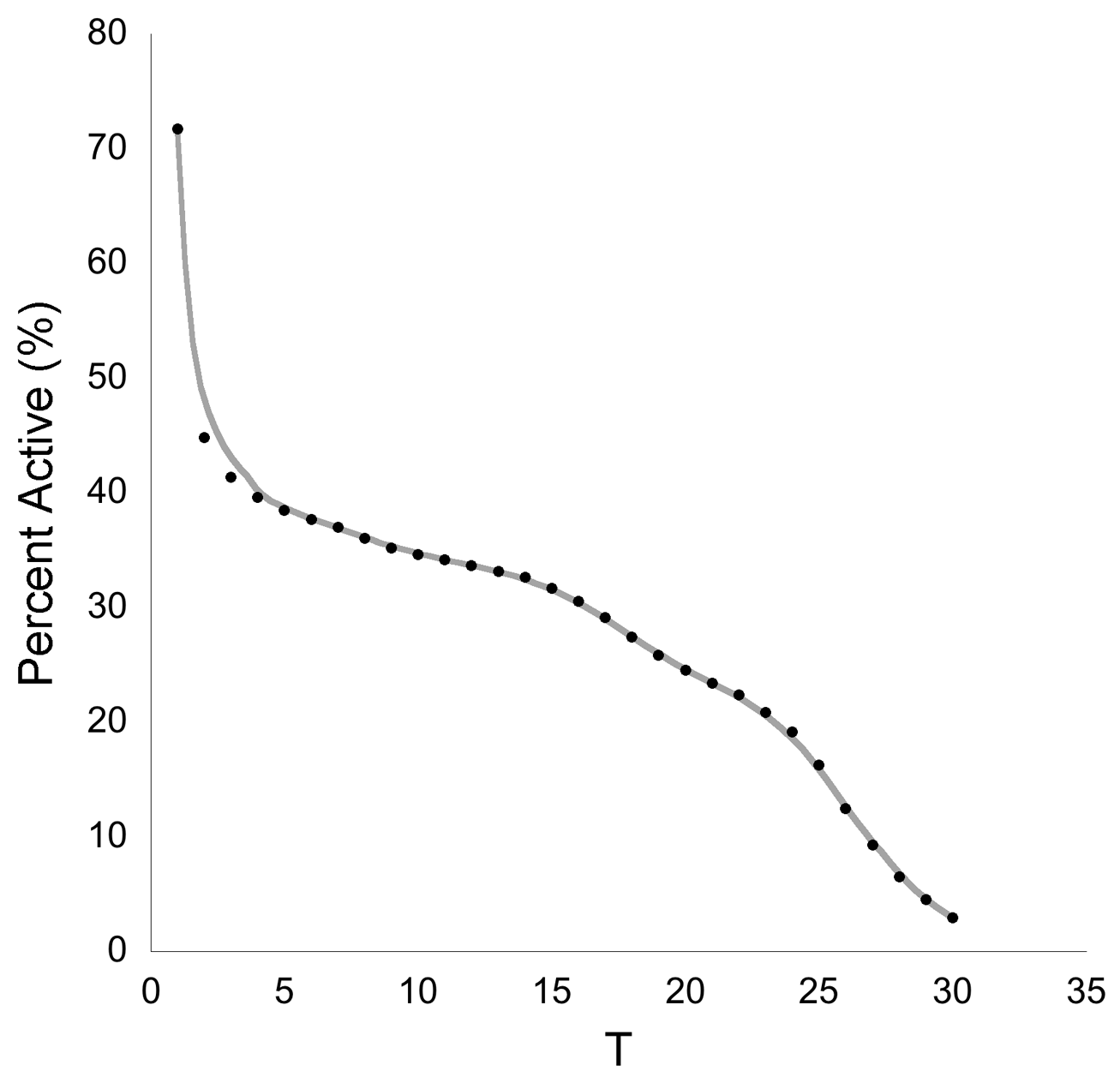

Supplement: Figure S1 — Perceived activity as a function of the threshold value. Percent active as a function of the threshold value (T) for badger N44 over the course of the study period. The threshold value chosen for this individual was 10. (TIF) [file pone.0083156.s001.tif]
