# Supplementary material for: Climate and the Individual: Inter-Annual Variation in the Autumnal Activity of the European Badger (Meles meles)
Source: PLoS One. 2014 Jan 17;9(1):e83156. doi: 10.1371/journal.pone.0083156 (PMC3894934; doi:10.1371/journal.pone.0083156)
Supplement: Appendix S1 — (DOCX) [file pone.0083156.s003.docx]

**Appendix S1**

Triaxial accelerometry provides a wealth of data about fine scale motion and can be used to infer behaviour and energetics. In this work we propose a method to determine how active an animal is, on a normalized scale with 0 (0%) corresponding to inactive and 1 (100%) corresponding to continuous activity. This method is scale-invariant and hence is useful to contrast the ratio of active to inactive time in animals.

**Approach**

**1)** Acceleration is sampled at 8 Hz on 3 axes simultaneously to provide a fine-scale record of animal behaviour.

**2)** The vector measurements are transformed to observed dynamic body acceleration (ODBA) as per the method in Wilson et al. (2006), here using a window length of 2 seconds to obtain the direction of *g*, followed by taking the L1 (absolute value) norm of the dynamic component.

**3)** ODBA is averaged over a 10 minute bin to give a metric of overall activity. In this way, brief spikes in acceleration, which are due to knocks and bumps, are filtered out, leaving the trend itself.

**4)** The mean ODBA is thresholded to determine if the animal was active (1) or inactive (0) during the window. Formally:

a_k_ = sgn(ODBA_k_ – T)

where a_k_ [0:1] is the activity in the kth window, sgn() is the signum function, ODBA_k_ is the time averaged ODBA value and T is a threshold value.

T should be chosen such that sampling noise and low-level motion from respiration is treated as being inactive and only dynamic motion that would contribute to energetic load above a baseline being regarding as being active. The value of T that best partitions the set into active and inactive subsets is determined using sensitivity analysis, a technique commonly used in robust control theory. Formally, this can be regarded as finding the value T that minimizes the gradient of the sensitivity function. Intuitively, if the threshold is too low, then the animal will be reported as being active 100% of the time. Conversely is the threshold is too high, then the animal will be reported as being active 0% of the time. The objective is to find an operating point where large changes in the threshold value lead to small changes in the overall duty cycle (ratio of active to inactive) (e.g., see Fig 1).
